# Phylogenetic Variants of *Rickettsia africae*, and Incidental Identification of "*Candidatus* Rickettsia Moyalensis" in Kenya

**DOI:** 10.1371/journal.pntd.0004788

**Published:** 2016-07-07

**Authors:** Gathii Kimita, Beth Mutai, Steven Ger Nyanjom, Fred Wamunyokoli, John Waitumbi

**Affiliations:** 1 Walter Reed Project/ Kenya Medical Research Institute, Kisumu, Kenya; 2 Department of Biochemistry, Jomo Kenyatta University of Agriculture and Technology, Juja, Kenya; Beijing Institute of Microbiology and Epidemiology, CHINA

## Abstract

**Background:**

*Rickettsia africae*, the etiological agent of African tick bite fever, is widely distributed in sub-Saharan Africa. Contrary to reports of its homogeneity, a localized study in Asembo, Kenya recently reported high genetic diversity. The present study aims to elucidate the extent of this heterogeneity by examining archived *Rickettsia africae* DNA samples collected from different eco-regions of Kenya.

**Methods:**

To evaluate their phylogenetic relationships, archived genomic DNA obtained from 57 ticks *a priori* identified to contain *R*. *africae* by comparison to *ompA*, *ompB* and *gltA* genes was used to amplify five rickettsial genes i.e. *gltA*, *ompA*, *ompB*, 17kDa and *sca4*. The resulting amplicons were sequenced. Translated amino acid alignments were used to guide the nucleotide alignments. Single gene and concatenated alignments were used to infer phylogenetic relationships.

**Results:**

Out of the 57 DNA samples, three were determined to be *R*. *aeschlimanii* and not *R*. *africae*. One sample turned out to be a novel rickettsiae and an interim name of “*Candidatus* Rickettsia moyalensis” is proposed. The bonafide *R*. *africae* formed two distinct clades. Clade I contained 9% of the samples and branched with the validated *R*. *africae str ESF-5*, while clade II (two samples) formed a distinct sub-lineage.

**Conclusions:**

This data supports the use of multiple genes for phylogenetic inferences. It is determined that, despite its recent emergence, the *R*. *africae* lineage is diverse. This data also provides evidence of a novel Rickettsia species, *Candidatus* Rickettsia moyalensis.

## Author Summary

*Rickettsia africae* is a bacterium mainly vectored by *Amblyomma* and *Rhipicephalus* species of ticks. It is the etiological agent of African tick bite fever (ATBF), a spotted fever rickettsiosis that presents as an acute febrile illness characterized by petecheal skin hemorrhages, from which the name is derived. This bacterium is probably the most important in sub-Saharan Africa, including Kenya, in terms of incidence and prevalence. This notwithstanding, the disease is poorly understood and is often mistreated as malaria, and therefore qualifies as a highly neglected disease. This study examined the genetic relationships of *R*. *africae* collected from diverse eco-regions of Kenya. We present data that indicate high genetic diversity in Kenya’s *R*. *africae* and corroborate a recent study that reported similar genetic diversity in *R*. *africae* samples collected from a localized area in western Kenya. Importantly, we describe a divergent lineage and propose the name *Candidatus* Rickettsia moyalensis.

## Introduction

Rickettsiae are obligate intracellular gram negative bacteria, belonging to the class *alpha-proteobacteria*. They are found in a diverse array of hosts ranging from vertebrates, arthropods, annelids, amoeba and plants. Based on a host perspective, the non-vertebrate-associated *Rickettsia* remain understudied and poorly characterized [1]. In contrast, the vertebrate-associated *Rickettsia* that are vectored by hematophagous arthropods such as ticks, fleas, lice and mites are better studied, are responsible for rickettsial diseases that are important cause of illness and death worldwide [2]. To date, this genus consists of 29 validated species and numerous partially characterized species [3,4], thus illustrating the difficulties of unravelling the composition of this seemingly homogeneous group of bacteria.

Phenotypic characters such as pathogenicity, growth temperature requirements, ability to polymerize host cell actin, and cross reactivity to somatic antigens of *Proteus vulgaris* strains (*OX19* and *OX2*) and *P*. *mirabilis OXK* have been used to infer evolutionary relationships amongst rickettsiae [5]. From these criteria, the genus *rickettsia* was organized into three bio-types, namely, spotted fever group (SFG), typhus group (TG) and scrub typhus group (STG). The phenotypic characters have been found to be unreliable estimators of their phylogeny [6]. The advent of molecular tools brought major reorganizations in *rickettsia* taxonomy. For instance, by analysis of *Rickettsia* 16S rRNA (*rrs*), STG was removed from the genus *Rickettsia* and placed into its own genus, *Orientia*. This genus currently has only two species, *O*. *tsutsugamushi* [7] and a recently described *O*. *chuto* [8].

Several genes have been used for *Rickettsia* phylogenetic systematics: the *rrs* [5], *gltA* [9], 17kDa [10], *ompA* [11], *ompB* [12], *sca4* [13], *sca2* [14], and more recently, complete genomic sequences [15,16]. Currently, the genus delineates into four clades [17–19]: *(i)* The SFG which is the most derived, and consists of 23 validated species including *R*. *africae*, and numerous partially characterized species. Using whole genome approach, it has been realized that some of the members of rickettsiae such as *R*. *helvetica* do not fit in the SFG [16,20]). *(ii)* The transitional group (TRG) whose members are *R*. *akari* and *R*. *felis*. *(iii)* The TG which has only two members, namely, *R*. *typhi* and *R*. *prowazekii*; and *(iv)*, the ancestral group (AG), whose members consists of *R*. *bellii* and *R*. *canadensis*. The use of the name TRG has been challenged [15,21]). In addition to the systemic of vertebrate-associated *Rickettsia*, clades associated with non-vertebrate *Rickettsia* have been described [16,19].

Of the rickettsiae, *R*. *africae* is probably the most important in Africa. It is the aetiological agent of African tick bite fever [22] and the most reported [23]. It has been reported in 22 sub-Saharan countries [3], the West Indies [24] and Oceania [25]. In Kenya, a recent surveillance study of rickettsiae in ticks identified 104 rickettsiae, of which 93% were *R*. *africae*, clearly, demonstrating its dominance [26]. *R*. *africae* is vectored by *Amblyomma* ticks, primarily *A*. *variegatum* and *A*. *hebraeum*. Infections have also been detected in many other ticks species [3,26–28] by PCR and not by competency studies.

In general, *Rickettsia* species are very closely related. Within the SFG where *R*. *africae* belongs, the mean nucleotide homogeneity with *rrs*, *gltA*, *ompA*, *ompB* and *sca4* genes ranges from 82.2% to 99.8% [29]. Considering this high interspecies homogeneity, intraspecies differences are even smaller. For example, using the more variable multi spacer typing that combined *dksA-xerC*, *mppA-purC*, and *rpmE-tRNAfMet* spacer sequences, it was impossible to discriminate *R*. *africae* strains [30]. Using *ompA* and *ompB* genes, many groups have however reported heterogeneity [25,31,32]. A recent study reported an even higher heterogeneity of *R*. *africae* samples collected from a localized area in Western Kenya [33]. The study reported here sought to determine how widespread the heterogeneity of Kenya’s *R*. *africae* is by examining DNA samples collected from different eco-regions of Kenya.

## Methods

### Ethics statement

This study was carried out using ticks collected from domestic animals presented for slaughter. The tick samples were collected under protocol SSC#1248 that was reviewed and approved by the Animal Use Committee of the Kenya Medical Research Institute.

### Sample acquisition and study site

Details of the areas the tick were collected from, method of collection, DNA extraction and preliminary genotyping have been published before [26] and are summarized in S1 Table.

### Amplification of target genes

Sequence data for 57 tick-extracted DNA samples that had been identified as *R*. *africae* by comparison to 385 bp citrate synthase (*gltA*) gene, 530 bp outer membrane protein A gene (*ompA*) and 444 bp outer membrane protein B gene (*ompB*) were obtained from our laboratory's database from a previous study [26]. The sequences of both strands were re-checked for correctness and errors cleaned. Samples with short or missing sequences were re-amplified and re-sequenced. Two additional genes: the 450 bp 17kDa and 2700 bp *sca4* genes were also amplified and sequenced.

Primers used to amplify target genes are listed in Table 1 and are previously described [34]. PCR reagents were obtained from Applied Biosystems (CA, USA) and reactions performed in a 25 μL reaction volume containing 10 μM of each primer, 200 μM of dNTP mix, 1.5U Taq polymerase and 2 mM MgCl_2_. Amplification was carried out in a DNA thermal cycler (HID Veriti) from Applied Biosystems (CA, USA). The following conditions were used for amplification: For *ompA* and *gltA* genes: 3 min of initial denaturation at 94°C, then 40 cycles at 94°C for 30 sec, 53°C for 30 sec, 68°C for 1 min. For the *ompB* gene: 3 min of initial denaturation at 95°C, then 40 cycles at 95°C for 30 sec, 50°C for 30 sec, 68°C for 1 min 30 sec. For the 17kDa gene: 5 min of initial denaturation at 94°C, then 35 cycles at 94°C for 30 sec, 50°C for 1 min, 68°C for 1 min. For *sca4*: 5 min of initial denaturation at 95°C, then 40 cycles at 95°C for 45 sec, 60°C for 30 sec and 68°C for 3 min. All the amplifications were then completed by holding for 7 min at 72°C. To ascertain correct product sizes, a portion of the amplicons (5μL) was run on a 1% (w/v) agarose gel containing ethidium bromide. Product sizes were estimated by comparing with a molecular mass standard (1kb plus ladder, Invitrogen, (CA, USA).

**Table 1:** Primers used for PCR and sequencing.

Gene	Oligo Sequence (5'-3')	Amplicon size
*gltA*	^1^^,^^2^CS49Fw: ACCTATACTTAAAGCAAGTATYGGT	385bp
	^1^CS1234Rv: TCTAGGTCTGCTGATTTTTTGTTCA	
	^2^CS1258Rv: ATTGCAAAAAGTACAGTGAACA	
*ompB*	^1^^,^^2^RAK1009Fw: ACATKGTTATACARAGTGYTAATGC	444bp
	^1^OmpB1902Rv: CCGTCATTTCCAATAACTAACTC	
	^2^RAK1452Rv: SGTTAACTTKACCGYTTATAACTGT	
*ompA*	^1^OmpAM50Fw: TTGCGTTATAACACTTTTTAAGTGA	530bp
	^1^OmpA642Rv: ATTACCTATTGTTCCGTTAATGGCA	
	^2^190-70Fw: ATGGCGAATATTTCTCCAAAA	
	^2^190-701Rv: GTTCCGTTAATGGCAGCATCT	
*sca4*	^1^D1Fw: ATGAGTAAAGACGGTAACCT	2700bp
	^1^D3069Rv: TCAGCGTTGTGGAGGGGAAG	
	^2^RrD749Fw: TGGTAGCATTAAAAGCTG	
	^2^RrD2685Rv: TTCAGTAGAAGATTTAGT	
17kDa	^1^Rp17kFw: AATGAGTTTTATACTTTACAAAATTCTAAAAACCA	450bp
	^1^^,^^2^Rr2608Rv: CATTGTTCGTCAGGTTGGCG	
	^2^Rr1175Fw: GCTCTTGCAACTTCTATGTT	

Fw = Foward primer; Rv = Reverse primer;

^1^ = primary PCR;

^2^ = secondary PCR and sequencing

### Gene sequencing

The PCR products were purified using Isolate II PCR and Gel Kit (Bioline, UK) as recommended by the manufacturer. The purified PCR products were sequenced in both directions using the Big Dye Terminator Cycle Sequencing Kit v 3.1 (Applied Biosystems, CA, USA) and the sequences analyzed by capillary electrophoresis in a 3130 Genetic Analyzer (Applied Biosystems). The sequences were proofread, edited and assembled into consensus sequences using CLC Main Workbench v 7 (CLC Inc, Aarhus, Denmark), and used to query GenBank using the nucleotide Basic Local Alignment Search Tool (BLAST) [35].

### Phylogenetic data analysis

Six different alignments were generated: Five of them corresponded to sequences of each target gene (*gltA*, *ompA*, *ompB*, 17kDa and *sca4*), and one corresponding to the concatenated sequence of all the five genes as well as the validated rickettsia strains derived from GenBank (see S2 Table for names of reference strains and their accession numbers). To ensure the accuracy of these alignments, nucleotide sequences were translated to their respective amino acids using the translate tools in the CLC Main Workbench v7. Amino acid alignments were made using Muscle v 3.8 software [36]. The protein alignments were then used to guide the corresponding nucleotide alignments using TOPALi V2 software [37].

For phylogenetic inference, MEGA v7 software [38] was used to estimate the best substitution model as well as estimate for the Maximum Likelihood (ML) trees for the individual genes. The concatenated alignment tree was also estimated with the ML method using a General Time Reversal (GTR) nucleotide substitution model with a gamma distribution (GTR+G). For bayesian probability analysis, jModeltest v 2.1 [39] was used to determine the GTR+G as the best fit model for *gltA*, *ompA*, *ompB*, 17kDa and *sca4* gene alignments. A partitioned analysis was then performed on the concatenated dataset using a bayesian Markov Chain Monte Carlo (MCMC) method implemented with MrBayes software v 3.2 [40]. The cluster confidence was given as posterior probabilities.

## Results

### Sample description

In total, 57 tick DNA samples were available for analysis. From these and as shown in supplementary information (S3 Table), 45 sequences were obtained for *gltA* (Genbank accession KX368721-KX368765), 57 for *ompA* (Accession: KX368868-KX368924), 44 for *ompB* (Accession: KX368823- KX368867), 57 for 17kDa (Accession: KX368766-KX368822) and 40 for *sca4* (Accession: KX368925-KX368964). The gene sequences were subjected to BLAST analysis for a preliminary verification of their identity. BLAST results are shown in S1 Table.

### Single gene topology trees and their ability to resolve study samples

**Fig 1:**
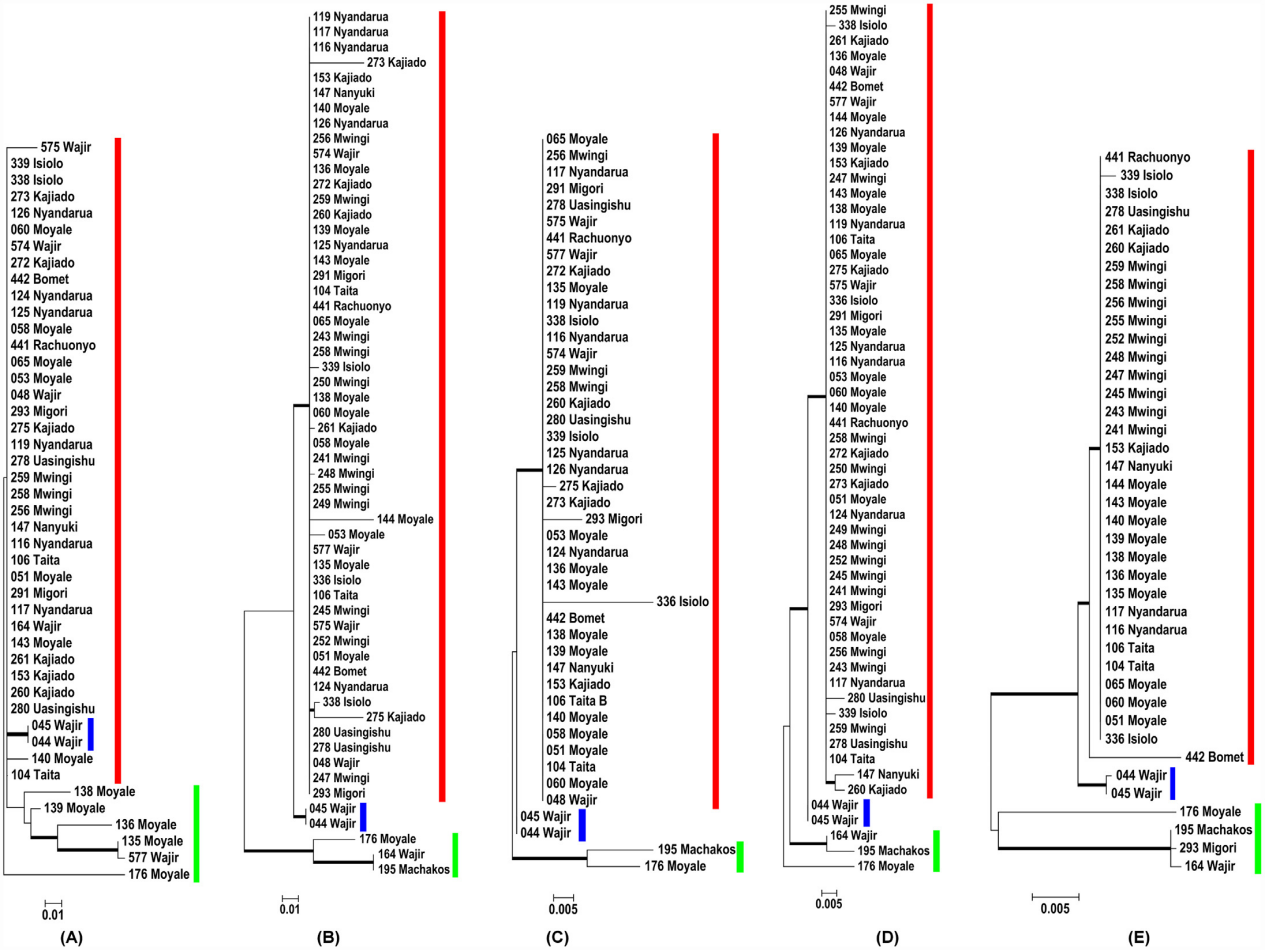
Phylogeny of Rickettsia study samples isolated from diverse eco-regions of Kenya. Maximum Likelihood trees were obtained from (A) *gltA*, (B) *ompA*, (C) *ompB*, (D) 17kDa and (E) *sca4* partial nucleotide sequences. Members of clade I, II and III are shown beside the bolded red, blue and green lines respectively. Numbers at the nodes are bootstrap proportions with 1000 replicates. Only bootstrap values >50% are shown. The scale bar indicates the number of substitutions per nucleotide position.

The topology of the *gltA* gene tree inferred with the ML method is shown in Fig 1, panel A. The tree shows unresolved evolutionary relationships (nodes with <50% bootstrap support values). Overlooking these clade credibility values, a major cluster (clade I) consisting of 89% of the sequences was observed in the more derived parts of the tree. The other two distinct clades were samples from the North Eastern part of Kenya. Clade II consisted of samples (*044* and *045* from Wajir) that diverged as sister operational taxonomic units (OTUs). Clade III samples clustered at the basal part of the tree and came from Moyale (*139*, *135*, *136 and 176*) and Wajir (*577*).

In comparison to *gltA*, the *ompA* tree resolved majority of the nodes into three well supported groups (Fig 1, panel B). As in *gltA*, clade I had the majority of OTUs (52/57, 91%), and was the most derived. Majority of the nodes were unresolved, and the members clustered in a polytomy. As in *gltA*, clade II formed a dichotomy made of samples *044* and *045* from Wajir. Clade III constituted a cluster of *176_Moyale*, *164_Waji*r and *195_Machakos* in the basal parts of the tree. In this clade, only *176_Moyale* was shared with the *gltA* gene tree.

As shown in Fig 1, panel C, *ompB* also resolved the study OTUs into three groups. Clade I contained 40/44 (91%) members in a polytomy. Clade II contained the same sister OTUs (*044 and 045)* from Wajir. Clade III had the same samples as in the *ompA* gene tree. As with *gltA*, *ompA* and *ompB* genes, the topology of the 17kDa gene tree was consistent in delineating three clades (Fig 1, panel D). Clade I contained 52/57 (91%) of unresolved OTUs. Clade II consisted of sister OTUs (*044* and *045*) from Wajir, while clade III consisted of *164_Wajir*, *195_Machakos* and *176_Moyale*

Compared to *gltA*, *ompA*, *ompB* and 17kDa, the *sca4* gene tree was better resolved especially in delineation of clade I (Fig 1, panel E) which consisted of 35/40 (86%) OTUs. Clade II consisted of sister OTUs (*044* and *045*) from Wajir. Clade III consisted of *176_Moyale*, *164_Wajir*, and an additional member *293_Migori*.

### Concatenating *gltA*, *ompA*, *ompB*, 17kDa and *sca4* genes allows better phylogenetic resolution

**Fig 2:**
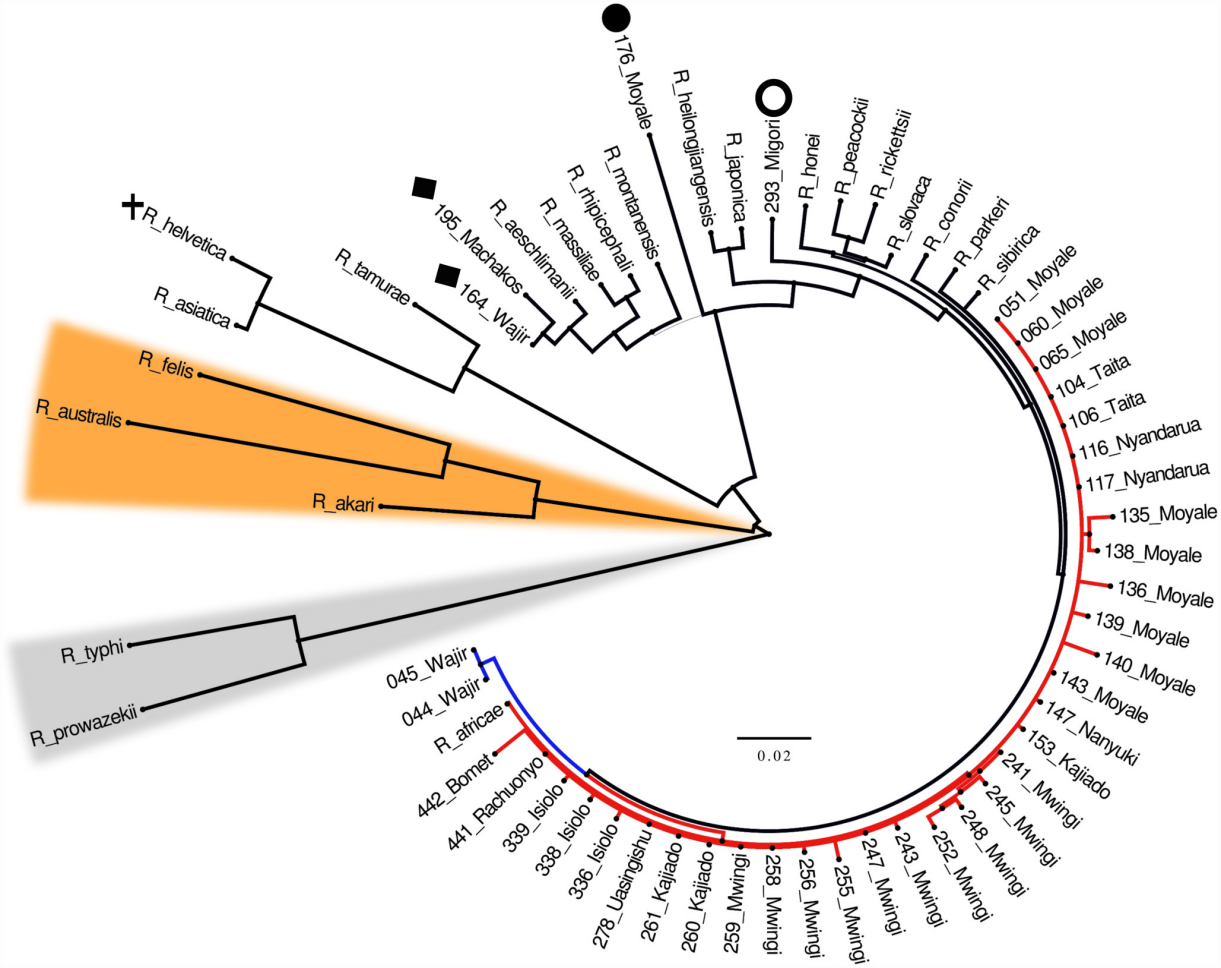
Bayesian probability tree of study samples with validated *Rickettsia* species. The tree is based on partitioned concatenated datasets of *gltA*, *ompA*, *ompB*, 17kDa and *sca4* partial nucleotide sequences. Amino acid alignments were used to guide the nucleotide alignments. The tree is estimated using a GTR+G substitution model as implemented in MrBayes v3.2. The tree is a consensus of 15,002 trees (post burn-in) pooled from two independent Markov Chain run in parallel. Thin lines indicate posterior probability values of < 1. Lineage diversity within the *R*. *africae* study samples is highlighted in red and blue to indicate clades i and ii respectively. Samples previously misclassified as *R*. *africae* are now classified as *R*. *aeschlimanii* (black diamond). Study sample 176_Moyale branches distinctly from other rickettsiae and is considered a novel rickettsia species and a provisional name "*Candidatus* rickettsia moyalensis" (black circle) is proposed. NB: Although 293_Migori (open circle) branched as a lone taxon, it clustered with *R*. *aeschlimanii* by Maximum Likelihood method. Non-spotted fever group lineages are highlighted orange for transition group and grey for typhus group. The status of *R*. *helvetica* (shown in black cross), originally in spotted fever group is now uncertain [20].

In order to improve the phylogenetic resolution of individual genes, the five genes were concatenated. Out of the 57 samples initially available for analysis, only 39 were included in the concatenation. The choice of the 39 was influenced by availability of the limiting gene (39 *sca4* samples), and not missing more than one of the other four gene sequences. The concatenated sequences also included validated Rickettsia species available in GenBank. The concatenated tree constructed with Bayesian method is shown in Fig 2. The validated *Rickettsia* sequences delineated into the three known *Rickettsia* clades: TG, TRG and SFG. All the 39 study OTUs lay within the SFG clade, of which 33/39 (84%) clustered with the validated *R*. *africae str ESF-5* shown as clade I. The two sister OTUs (*044* and *045*) from Wajir formed a distinct clade (clade II) that shared the most recent common ancestor with clade I. Two other samples (*164_Wajir* and *195_Machakos*) previously identified as *R*. *africae* [26] delineated with the validated *R*. *aeschlimanii*. The positions of these OTUs held when tested by ML method (S1 Fig).

The position of *293_Migori* was however tenuous, as it branched with *R*. *aeschlimanii* on ML method, but as a lone taxon between *R*. *heilongjiangensis* and *R*. *slovaca* on Bayesian analysis. Another interesting sample *176_Moyale* branched as a lone taxon with total statistical support (a posterior probability value of 1), thus raising questions concerning its taxonomic status. We consider this sample as a novel rickettsiae and an interim name of “*Candidatus* Rickettsia Moyalensis” is proposed.

### Phylogeny of R. africae study OTUs and those collected previously in Kenya

**Fig 3:**
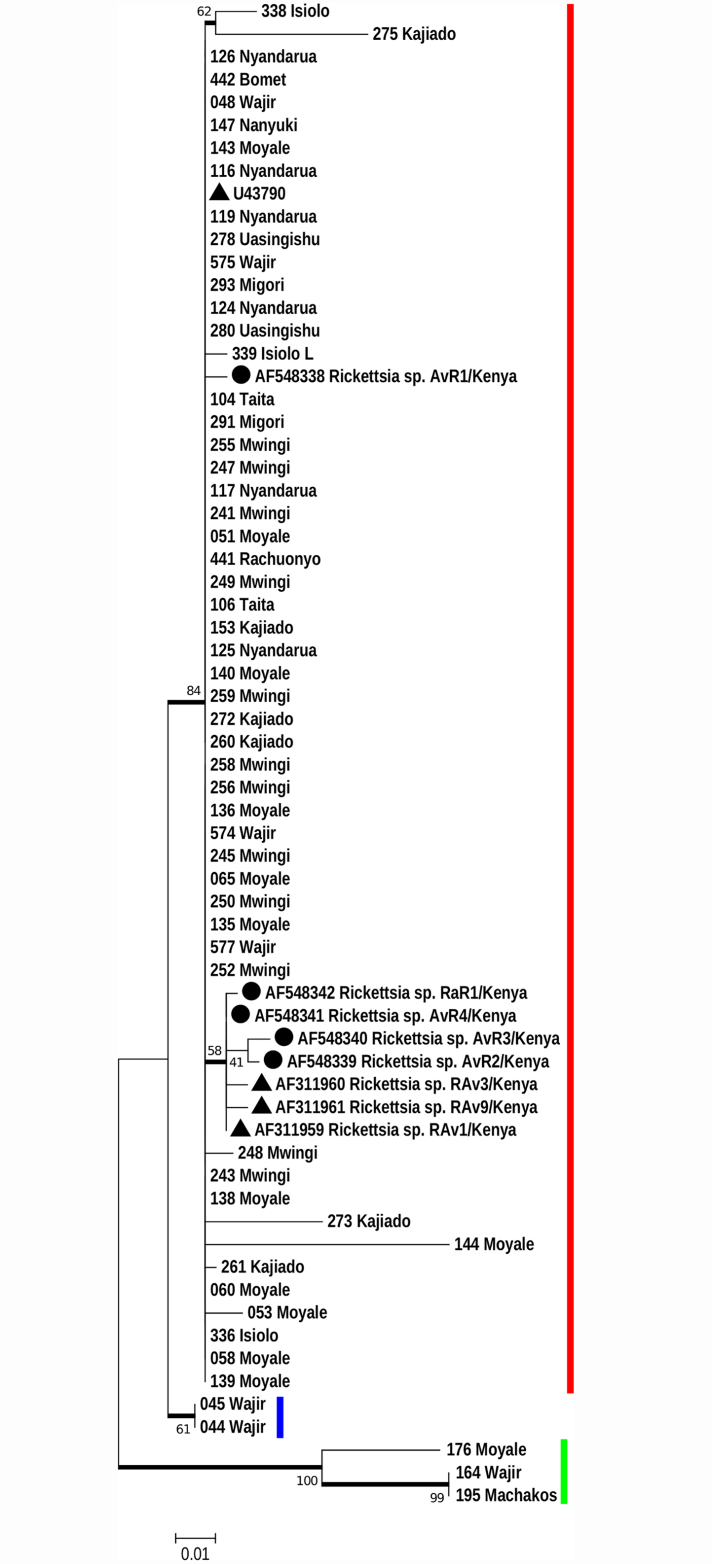
Phylogeny of Rickettsia sequences from this study and those collected previously in Kenya [23, 31]. *ompA* nucleotide sequences of study isolates and other *R*. *africae* reported from previous studies [23,31] were analysed by Maximum Likelihood method using MEGA v7 based on the Hasegawa-Kishino-Yano (HKY) model of substitution. The tree has a log likelihood ratio of -1049 and involved all codon positions. Members of clade I, II and III are shown beside the bolded red, blue and green lines respectively. Sequences from Parola et al 2001 [23] are shown as black triangles and those from Macaluso et al 2003 [31] by black circles. Numbers at the nodes are bootstrap proportions with 1000 replicates. Only bootstrap values >50% are shown. The scale bar indicates the number of substitutions per nucleotide position. Clearly, five of our sequences (044, 045 and 164 from Wajir, 176 Moyale and 195 Machakos) are distinct from those described previously.

The *ompA* gene sequences obtained from our study samples and those published previously collected in Kenya [23,31] were used to generate a phylogenetic tree (Fig 3). With this tree, three clades were discernible. Clade I contained majority of the OTUs 61/66 (92.4%) and a cluster of other 7 that formed a subclade within clade I, that consisted of OTUs published in previous studies. Clade II consisted of sister OTUs (044 and 045) from Wajir, and a basal clade III that consisted of members *176_Moyale*, *164_Wajir*, and *195_Machakos*. Clearly, five of our sequences (*044*, *045* and *164* from *Wajir*, *176 Moyale* and *195 Machakos*) are distinct from those described previously.

## Discussion

In this study, a combination of relatively conserved (*gltA*, 17kD) and variable *(ompA*, *ompB*, *and sca4)* genes were used to infer the evolutionary topology of *R*. *africae* using DNA samples obtained from ticks [26]. This work expands on a recent study that reported a significant heterogeneity of *R*. *africae* samples collected from a localized area in Western Kenya [33]. We also report on possible existence of a sub-lineage within the *R*. *africae* samples, as well as identify a putative novel *Rickettsia* species that was associated with *R*. *appendiculatus* tick collected from a cow in Moyale County, in Northern Kenya.

Due to its intracellular lifestyle, rickettsiae are highly dependent on their primary tick vectors and tend to be selective for the tick species they infect. For example, *R*. *africae* are primarily vectored by the *Amblyomma* species [41] but infections have also been found in *Rhipicephalus* and *Hyalomma* ticks [3]. As shown in S1 Table, 84% of the *R*. *africae* sequences came from *Amblyomma* and *Rhipicephalus* ticks. The remaining 16% came from *H*. *truncatum* and other unspeciated *Hyalomma* ticks. Nevertheless, without competency studies, it is difficult to say which of these findings were true infections.

The *gltA* gene codes for citrate synthase, an enzyme ubiquitously found in nearly all living cells, and is central in energy metabolism [42]. Evolutionary history inferred from this gene demonstrated low sequence divergence and yielded poorly supported clades with unresolved nodes (Fig 1, panel A). This is expected considering that, even within the *Rickettsia* genus, *gltA* gives poor interspecies resolution especially for the more derived branches of the SFG [9]. Nevertheless, three unsupported groupings (Fig 1, panel A, shown by red, blue and green lines) were discernible. It could be argued that the lack of resolution emanated from using a small fragment (385 bp) compared to 1234 bp that was used by Roux et al [9]. The Roux study aimed to develop *gltA* gene as a phylogenetic marker for Rickettsia species. From their generated phylogenies, the resolution decreased within the more recently emerged species, of which *R*. *africae* is a member. Our study that focused on intra-species variation within the *R*. *africae* lineage had similar problems with *gltA* and it is doubtful that a longer *glt*A that failed to resolve the recently evolved rickettsiae would have resolved variation within the study OTUs. We think longer fragments may have increased the number of variable characters within the gene but not the resolution.

As shown in Fig 1 (panels B, C and D), gene trees derived from *ompA*, *ompB* and 17kDa had very similar topologies and the three groupings seen in *gltA* tree were better supported. This topological concordance gives credence to the derived gene trees. By BLAST analysis, majority of the OTUs clustering in clade I were identical to *R*. *africae* reference strain ESF (S1 Table).

Tree topologies derived from individual genes identified clades II and III as outliers (Fig 1, blue and green lines). Interestingly, for all the genes, clade II had only two members (*044_Wajir* and *045_Wajir*) that appeared as a sub-lineage within *R*. *africae*. With four of the five genes analysed, BLAST analysis of the two samples showed highest identity to *R*. *africae* (accession no. HQ335126) with 99% for *gltA*, >99% for *ompA* (accession no. HQ335132), 99% for 17kDa (accession no. KF646137) and 100% for *sca4* (accession no. CP001612). BLAST analysis of the *ompB* gene showed highest homology (98%) to *R*. *mongolotimonae* (accession no. DQ097083). Within our study OTUs, members of clade III were the most genetically distant. With the *Sca4*, one member (*293_Migori*) that had consistently been in clade I was placed in clade III.

From the foregoing, the limitations of gene trees constructed from single genes are evident and give credence to recommendations to use a variety of genes sampled from different regions of the genome as the best practice for phylogeny assignment [43]. Fig 2 shows a Bayesian tree of study OTUs and the validated *Rickettsia* species derived from a concatenated sequence of *gltA*, *ompA*, *ompB*, 17kDa *and sca4*. The concatenated tree confirms the branching orders of clade I and authenticates members of clade II (*044_ and 045_Wajir*) as being a sub-lineage of *R*. *africae*. This derivation was confirmed by ML method (S1 Fig). Only after concatenation is the picture clearer that, the majority of members populating clade III in single gene analysis are not *R*. *africae*. Samples *164_Wajir* and *195_Machakos* (both associated with *H*. *truncatum* ticks from a cow) delineate with *R*. *aeschlimanii*, while *293_Migori* appeared as a lone taxon (Fig 2).

Since sample *176_Moyale* did not branch with any of the validated species in the concatenated tree (Fig 2), its sequence was compared to isolates available in GenBank. With the 276 bp *gltA* gene, it was most identical to *R*. *heilongjiangensis* (97.0%). With this gene, a homology of 99.9% is required in order for the sample to qualify as *R*. *heilongjiangensis* [29]. With the 489 bp fragment of *ompA* gene, the DNA was most identical to *Candidatus* R. amblyommii (97.0%) against the required homology of 98.8%. With the *ompB* gene, the 267 bp fragment was most identical to *Rickettsia rhipicephali* (99.0%) against a required homology of 99.2% to qualify as *R*. *rhipicephali*. With the *sca4* gene, the 1846 bp was most similar to *R*. *africae str ESF5* (97.0%) against the required homology of 99.3%. For the 17kDa, the closest homology was with *Rickettsia raoultii str Khabarovsk* (97.0%) identity. There are no published homology requirements for 17kDa gene. Given this level of nucleotide sequence divergence from validated *Rickettsia* species, our results support the consideration of *176_Moyale* as a sample from a new *Rickettsia* species. Until grown in culture, and its biology elucidated, we propose that this sample be identified as “*Candidatus* R. moyalensis”.

Two other studies in Kenya have reported genetic heterogeneity within the *R*. *africae* lineage, one in Maasai Mara game reserve [31] and another in rural farming community in Asembo, Nyanza province [33]. The current study extends these findings and identifies Northern Kenya (Moyale and Wajir) as harbouring more heterogeneous *R*. *africae* or completely new species (Fig 2 and S1 Fig) compared to other regions. To determine homology of our study OTUs and those described as *R*. *africae* variants in previous studies in Kenya [23, 31], *ompA* gene sequences were compared. As shown in Fig 3, five of our sequences (*044*, *045* and *164* from *Wajir*, *176 Moyale* and *195 Machakos*) are distinct from those described previously. Unfortunately, we did not sequence a second region of *ompA* and *ompB* genes that were associated with significant sequence variation [33]. The Rickettsia species dynamics in Kenya are probably being moulded by: 1) the highly mobile nomadic populations that move across wide geographical borders that create opportunities for mixing of rickettsiae from different livestock species (goats, sheep, cows, donkeys and camels). 2) The encroachment of wildlife habitats by nomadic pastoralists that introduce previously isolated *Rickettsia* species in wildlife to livestock. We speculate that these factors could be responsible for the observed genetic variations within the Kenyan *R*. *africae*, as well providing opportunity for genetic mixing that may result in creation of new lineages.

### Conclusion

This study provides new information regarding the phylogenetic relationships of the *R*. *africae* lineage. This lineage, though only recently emerged [30] is clearly undergoing diversification, as observed in the branching order of the samples studied. A definite sub-lineage of *R*. *africae* (samples 044 and 045 from Wajir) was identified. It was impossible to confirm the placement of sample 293_Migori. Additional sequence data will be required to resolve the ambiguity. Lastly, a putative novel rickettsiae (sample 176_Moyale) with a proposed name of “*Candidatus* Rickettsia moyalensis” was identified. Further work will be needed to determine its prevalence in Kenya and its implications to human and/or animal disease.

## Supporting Information

S1 Table *Rickettsia* samples used in the study. (DOCX)

S2 Table Names and accession numbers for validated strains used in this study. (DOCX)

S3 Table List of Genbank accession numbers of study OTUs. (DOCX)

S1 Fig Maximum Likelihood tree of study samples and validated *Rickettsia* species. A General Time Reversal with Gamma distribution (GTR+G) model was used to infer phylogeny of concatenated partial sequences of *gltA*, *ompA*, *ompB*, 17kDa and *sca4* nucleotide sequences. Amino acid alignments were used to guide the nucleotide alignments. The tree with the highest log likelihood (-8252.6475) is shown. Study samples that are bonafide *R*. *africae* aggregate in clades I and II. Samples previously misclassified as *R*. *africae* are now classified as *R*. *aeschlimanii* (black diamond). Study sample *176_Moyale* branches distinctly from other rickettsiae and is considered a novel rickettsia species provisionally named "*Candidatus* rickettsia moyalensis" (black circle). With this method, 293_Migori (open circle) clusters with *R*. *aeschlimanii*. Numbers at the nodes are bootstrap proportions with 1000 replicates. Only bootstrap values >50% are shown. SFG = spotted fever group, TRG = transition group, TG = typhus group. The status of *R*. *helvetica* (shown in black cross), originally in spotted fever group is now uncertain [20].
(TIF)
