# Supplementary material for: Phylogenetic Variants of Rickettsia africae, and Incidental Identification of "Candidatus Rickettsia Moyalensis" in Kenya
Source: PLoS Negl Trop Dis. 2016 Jul 7;10(7):e0004788. doi: 10.1371/journal.pntd.0004788 (PMC4936727; doi:10.1371/journal.pntd.0004788)
Supplement: S1 Table — (DOCX) [file pntd.0004788.s001.docx]

**S1 Table: *Rickettsia* samples used in the study**

| **Animal source** | **Tick sample** | **County of origin** | **Tick Source** | **% Sequence homology results with BLAST analysis.** | | | | |
| --- | --- | --- | --- | --- | --- | --- | --- | --- |
|  |  |  |  | ***gltA*** | ***ompA*** | ***ompB*** | **17kDa** | ***sca4*** |
| Goat | 44 | Wajir | *Rhipicephalas pulchellus* | \| HQ335126 \| \| --- \|   99% | HQ335132 100% | DQ097083 100% | KF646137 99% | CP001612 100% |
|  | 45 | Wajir | *Hyalomma sp* | HQ335126 99% | HQ335132 99% | DQ097083 99% | KF646137 99% | CP001612 100% |
|  | 48 | Wajir | *R. pulchellus* | KJ410266 99% | GU247115 99% | CP001612 100% | CP001612 99% | NA |
| Sheep | 147 | Nanyuki | *R. pulchellus* | KJ941102 100% | GU247115 99% | CP001612 98% | CP001612 98% | CP001612 100% |
|  | 153 | Kajiado | *R. pulchellus* | KJ941102 99% | GU247115 100% | CP001612 100% | CP001612 100% | CP001612 99% |
|  | 260 | Kajiado | *A. gemma* | KJ941102 99% | AB934397 100% | NA | CP001612 97% | CP001612 99% |
|  | 261 | Kajiado | *R. pulchellus* | KJ941102 98% | GU247115 100% | NA | CP001612 99% | CP001612 99% |
|  | 272 | Kajiado | *R. appendiculatus* | GU131156 100% | AB934397 99% | CP001612 100% | CP00161299% | NA |
|  | 273 | Kajiado | *H. truncatum* | GU131156 100% | GU247115 89% | CP001612 99% | CP001612 99% | NA |
|  | 275 | Kajiado | *H. truncatum* | KJ410266 99% | GU247115 89% | CP001612 99% | CP001612 100% | NA |
| Cow | 51 | Moyale | *Amblyomma hebraeum* | KJ941102 100% | GU247115 100% | CP001612 99% | CP001612 98% | CP001612 99% |
|  | 53 | Moyale | *R. appendiculatus* | KJ410266 99% | AB934397 98% | CP001612 98% | CP001612 100% | NA |
|  | 58 | Moyale | *R. pulchellus* | GU131156 99% | AB822462 100% | CP001612 100% | CP001612 98% | NA |
|  | 60 | Moyale | *A. hebraeum* | GU131156 100% | JN043509 100% | CP001612 98% | CP001612 100% | CP001612 100% |
|  | 65 | Moyale | *R. pulchellus* | KJ410266 99% | GU247115 99% | CP001612 100% | CP001612 100% | CP001612 100% |
|  | 104 | Taita | *A. hebraeum* | KJ941102 96% | GU247115 100% | CP001612 99% | CP001612 98% | CP001612 99% |
|  | 106 | Taita | *A. gemma* | KJ941102 100% | GU247115 100% | CP001612 99% | CP001612 100% | CP001612 100% |
|  | 116 | Nyandarua | *A. gemma* | KJ941102 100% | GU247115 100% | CP001612 100% | CP001612 100% | CP001612 100% |
|  | 117 | Nyandarua | *Hyalomma truncatum* | KJ410266 99% | GU247115 100% | CP001612 100% | CP001612 99% | CP001612 100% |
|  | 119 | Nyandarua | *R. annulatus* | KJ410266 99% | GU247115 100% | CP001612 100% | CP001612 100% | NA |
|  | 124 | Nyandarua | *R. annulatus* | GU131156 99% | GU247115 100% | CP001612 99% | CP001612 100% | NA |
|  | 125 | Nyandarua | *R. annulatus* | KJ410266 99% | GU247115 100% | CP001612 99% | CP001612 100% | NA |
|  | 126 | Nyandarua | *H. truncatum* | GU131156 100% | GU247115 99% | CP001612 99% | CP001612 100% | NA |
|  | 135 | Moyale | *A. hebraeum* | KJ941102 98% | GU247115 98% | CP001612 100% | CP001612 100% | CP001612 100% |
|  | 136 | Moyale | *A. gemma* | KJ941102 98% | GU247115 99% | CP001612 99% | CP001612 99% | CP001612 100% |
|  | 138 | Moyale | *A. hebraeum* | KJ941102 97% | GU247115 97% | CP001612 99% | CP001612 100% | CP001612 100% |
|  | 139 | Moyale | *A. gemma* | KJ941102 99% | AB934397 99% | CP001612 98% | CP001612 100% | CP001612 100% |
|  | 140 | Moyale | *A. gemma* | DQ423368 96% | GU247115 99% | CP001612 97% | CP001612 99% | CP001612 100% |
|  | 143 | Moyale | *A. hebraeum* | AY737684 99% | GU247115 99% | CP001612 99% | CP001612 100% | CP001612 100% |
|  | 144 | Moyale | *A. gemma* | NA | GU247115 97% | NA | CP001612 99% | CP001612 100% |
|  | 164 | Wajir | *H. truncatum* | KJ663742 100% | KJ663748 100% | NA | KF646135 98% | HM05275 100% |
|  | 176 | Moyale | *R. appendiculatus* | JX945522 96% | KF702333 97% | CP013133 98% | CP010969 97% | CP001612 97% |
|  | 195 | Machakos | *H. truncatum* | NA | KJ663748 100% | AF123705 97% | KF646135 100% | HM050275 99% |
|  | 241 | Mwingi | *A. gemma* | NA | GU247115 100% | NA | CP001612 99% | CP001612 100% |
|  | 243 | Mwingi | *R. pulchellus* | NA | GU247115 99% | NA | CP001612 99% | CP001612 100% |
|  | 245 | Mwingi | *A. hebraeum* | NA | GU247115 99% | NA | CP001612 97% | CP001612 100% |
|  | 247 | Mwingi | *A. gemma* | NA | GU247115 100% | NA | CP001612 100% | CP001612 100% |
|  | 248 | Mwingi | *A. hebraeum* | NA | GU247115 99% | NA | CP001612 98% | CP001612 100% |
|  | 249 | Mwingi | *A. gemma* | NA | GU247115 100% | NA | CP001612 98% | NA |
|  | 250 | Mwingi | *A. hebraeum* | NA | GU247115 100% | NA | CP001612 98% | NA |
|  | 252 | Mwingi | *A. gemma* | NA | AB822460 96% | NA | CP001612 99% | CP001612 100% |
|  | 255 | Mwingi | *R. pulchellus* | NA | GU247115 100% | NA | CP001612 99% | CP001612 100% |
|  | 256 | Mwingi | *A. gemma* | NA | GU247115 99% | CP001612 99% | CP001612 99% | CP001612 100% |
|  | 258 | Mwingi | *A. gemma* | NA | JN043509 100% | CP001612 100% | CP001612 99% | CP001612 100% |
|  | 259 | Mwingi | *R. pulchellus* | NA | AB934397 100% | CP001612 98% | CP001612 99% | CP001612 100% |
|  | 278 | Uasingishu | *H. truncatum* | NA | GU247115 100% | CP001612 100% | CP001612 98% | CP001612 100% |
|  | 280 | Uasingishu | *H. truncatum* | NA | GU247115 100% | CP001612 99% | CP001612 99% | NA |
|  | 291 | Migori | *R. annulatus* | KJ410266 99% | GU247115 100% | CP001612 100% | CP001612 100% | NA |
|  | 293 | Migori | *A. variegatum* | KJ410266 99% | GU247115 100% | CP001612 99% | CP001612 99% | HM050275 98% |
|  | 336 | Isiolo | *A hebraeum* | NA | GU247115 98% | CP001612 98% | CP001612 100% | CP001612 100% |
|  | 338 | Isiolo | *A gemma* | GU131156 100% | GU247115 98% | CP001612 100% | CP001612 100% | CP001612 100% |
|  | 339 | Isiolo | *R. pulchellus* | GU131156 100% | AB822471 100% | CP001612 99% | CP001612 99% | CP001612 99% |
|  | 441 | Rachuonyo | *R. appendiculatus* | GU131156 99% | GU247115 100% | CP001612 100% | CP001612 99% | CP001612 100% |
|  | 442 | Bomet | *A. hebraeum* | GU131156 99% | GU247115 98% | CP001612 99% | CP001612 99% | CP001612 100% |
|  | 574 | Wajir | *R. annulatus* | NA | GU247115 99% | CP001612 100% | CP001612 99% | NA |
|  | 575 | Wajir | *A. hebraeum* | HM050296 96% | GU247115 100% | CP001612 100% | CP001612 100% | NA |
|  | 577 | Wajir | *A. variegatum* | KJ941102 97% | GU247115 94% | CP001612 100% | CP001612 99% | NA |

NA=Sequence not Available
