# Supplementary material for: Phylogenetic Variants of Rickettsia africae, and Incidental Identification of "Candidatus Rickettsia Moyalensis" in Kenya
Source: PLoS Negl Trop Dis. 2016 Jul 7;10(7):e0004788. doi: 10.1371/journal.pntd.0004788 (PMC4936727; doi:10.1371/journal.pntd.0004788)
Supplement: S2 Table — (DOCX) [file pntd.0004788.s002.docx]

**S2 Table: Accession numbers for validated strains used in this study**

| **Species (validated)** | **Strain** | **Genome (Accession no).** | **GenBank accession no** | | | | |
| --- | --- | --- | --- | --- | --- | --- | --- |
|  |  |  | ***gltA*** | ***ompA*** | ***ompB*** | ***17kD*** | ***sca4*** |
| *R. prowazekii* | *Breinl* | NC_020993 | † | NA | † | † | † |
| *R. typhi* | *Wilmington* | NC_006142 | † | NA | † | † | † |
| *R. helvetica* | *C9P9* | NZ_CM001467 | † | NA | † | † | † |
| *R. rickettsii* | *Sheila Smith* | CP000848 | † | † | † | † | † |
| *R. conorii* | *Seven (Malish)* | AE006914 | † | † | † | † | † |
| *R. africae* | *ESF-5* | CP001612 | † | † | † | † | † |
| *R. sibirica* | *246* | NZ_AABW01000001 | † | † | † | † | † |
| *R. honei* | *TT118* | ‡ | U59726 | U43809 | AF123724 | AF027124 | AF163004 |
| *R. slovaca* | *13B* | NC_016639 | † | † | † | † | † |
| *R. parkeri* | *Portsmouth* | NC_017044 | † | † | † | † | † |
| *R. japonica* | *YH* | AP011533 | † | † | † | † | † |
| *R. akari* | *Hartford* | NC_009881 | † | † | † | † | † |
| *R. australis* | *Cutlack* | NC_017058 | † | † | † | † | † |
| *R. felis* | *URRWXCal2* | NC_007109 | † | † | † | † | † |
| *R. massiliae* | *MTU5* | CP000683 | † | † | † | † | † |
| *R. montanensis* | *OSU* | NC_017043 | † | † | † | † | † |
| *R. rhipicephali* | *3-6-7* | NC_017042 | † | † | † | † | † |
| *R. aeschlimannii* | *MC16T* | ‡ | U59722 | U43800 | AF123705 | NA | AF163005 |
| *R. asiatica* | IO-1 | ‡ | AF394901 | NA | DQ110870 | AB114798 | DQ110869 |
| *R. tamurae* | AT-1 | ‡ | AF394896 | DQ103259 | DQ113910 | AB114825 | DQ113911 |
| *R. heilongiangensis* | 054 | NC_015866 | † | † | † | † | † |
| *R. peacockii* | *Rustic* | NC_012730 | † | † | † | † | † |
|  |  |  |  |  |  |  |  |

†= Complete genome available in Genbank

‡= Individual genes sequences available in the Genbank

NA= Gene sequences not available in the Genbank
