# Supplementary material for: Phylogenetic Variants of Rickettsia africae, and Incidental Identification of "Candidatus Rickettsia Moyalensis" in Kenya
Source: PLoS Negl Trop Dis. 2016 Jul 7;10(7):e0004788. doi: 10.1371/journal.pntd.0004788 (PMC4936727; doi:10.1371/journal.pntd.0004788)
Supplement: S3 Table — (DOCX) [file pntd.0004788.s003.docx]

**S3 Table: List of Genbank accession numbers of study OTUs**

| GENE | SEQ | ACCESSION |
| --- | --- | --- |
| *gltA* | 044_Wajir | KX368721 |
| *gltA* | 045_Wajir | KX368722 |
| *gltA* | 048_Wajir | KX368723 |
| *gltA* | 051_Moyale | KX368724 |
| *gltA* | 053_Moyale | KX368725 |
| *gltA* | 058_Moyale | KX368726 |
| *gltA* | 060_Moyale | KX368727 |
| *gltA* | 065_Moyale | KX368728 |
| *gltA* | 104_Taita | KX368729 |
| *gltA* | 106_Taita | KX368730 |
| *gltA* | 116_Nyandarua | KX368731 |
| *gltA* | 117_Nyandarua | KX368732 |
| *gltA* | 119_Nyandarua | KX368733 |
| *gltA* | 124_Nyandarua | KX368734 |
| *gltA* | 125_Nyandarua | KX368735 |
| *gltA* | 126_Nyandarua | KX368736 |
| *gltA* | 135_Moyale | KX368737 |
| *gltA* | 136_Moyale | KX368738 |
| *gltA* | 138_Moyale | KX368739 |
| *gltA* | 139_Moyale | KX368740 |
| *gltA* | 140_Moyale | KX368741 |
| *gltA* | 143_Moyale | KX368742 |
| *gltA* | 147_Nanyuki | KX368743 |
| *gltA* | 153_Kajiado | KX368744 |
| *gltA* | 164_Wajir | KX368745 |
| *gltA* | 176_Moyale | KX368746 |
| *gltA* | 256_Mwingi | KX368747 |
| *gltA* | 258_Mwingi | KX368748 |
| *gltA* | 259_Mwingi | KX368749 |
| *gltA* | 260_Kajiado | KX368750 |
| *gltA* | 261_Kajiado | KX368751 |
| *gltA* | 272_Kajiado | KX368752 |
| *gltA* | 273_Kajiado | KX368753 |
| *gltA* | 275_Kajiado | KX368754 |
| *gltA* | 278_Uasingishu | KX368755 |
| *gltA* | 280_Uasingishu | KX368756 |
| *gltA* | 291_Migori | KX368757 |
| *gltA* | 293_Migori | KX368758 |
| *gltA* | 338_Isiolo | KX368759 |
| *gltA* | 339_Isiolo | KX368760 |
| *gltA* | 441_Rachuonyo | KX368761 |
| *gltA* | 442_Bomet | KX368762 |
| *gltA* | 574_Wajir | KX368763 |
| *gltA* | 575_Wajir | KX368764 |
| *gltA* | 577_Wajir | KX368765 |
|  |  |  |
| 17kDa | 044_Wajir | KX368766 |
| 17kDa | 045_Wajir | KX368767 |
| 17kDa | 051_Moyale | KX368768 |
| 17kDa | 053_Moyale | KX368769 |
| 17kDa | 058_Moyale | KX368770 |
| 17kDa | 060_Moyale | KX368771 |
| 17kDa | 065_Moyale | KX368772 |
| 17kDa | 104_Taita | KX368773 |
| 17kDa | 106_Taita | KX368774 |
| 17kDa | 116_Nyandarua | KX368775 |
| 17kDa | 117_Nyandarua | KX368776 |
| 17kDa | 119_Nyandarua | KX368777 |
| 17kDa | 124_Nyandarua | KX368778 |
| 17kDa | 125_Nyandarua | KX368779 |
| 17kDa | 126_Nyandarua | KX368780 |
| 17kDa | 135_Moyale | KX368781 |
| 17kDa | 136_Moyale | KX368782 |
| 17kDa | 138_Moyale | KX368783 |
| 17kDa | 139_Moyale | KX368784 |
| 17kDa | 140_Moyale | KX368785 |
| 17kDa | 141_Moyale | KX368786 |
| 17kDa | 143_Moyale | KX368787 |
| 17kDa | 144_Moyale | KX368788 |
| 17kDa | 147_Nanyuki | KX368789 |
| 17kDa | 153_Kajiado | KX368790 |
| 17kDa | 164_Wajir | KX368791 |
| 17kDa | 176_Moyale | KX368792 |
| 17kDa | 195_Machakos | KX368793 |
| 17kDa | 241_Mwingi | KX368794 |
| 17kDa | 243_Mwingi | KX368795 |
| 17kDa | 245_Mwingi | KX368796 |
| 17kDa | 247_Mwingi | KX368797 |
| 17kDa | 248_Mwingi | KX368798 |
| 17kDa | 249_Mwingi | KX368799 |
| 17kDa | 250_Mwingi | KX368800 |
| 17kDa | 252_Mwingi | KX368801 |
| 17kDa | 255_Mwingi | KX368802 |
| 17kDa | 256_Mwingi | KX368803 |
| 17kDa | 258_Mwingi | KX368804 |
| 17kDa | 259_Mwingi | KX368805 |
| 17kDa | 260_Kajiado | KX368806 |
| 17kDa | 261_Kajiado | KX368807 |
| 17kDa | 272_Kajiado | KX368808 |
| 17kDa | 273_Kajiado | KX368809 |
| 17kDa | 275_Kajiado | KX368810 |
| 17kDa | 278_Uasingishu | KX368811 |
| 17kDa | 280_Uasingishu | KX368812 |
| 17kDa | 291_Migori | KX368813 |
| 17kDa | 293_Migori | KX368814 |
| 17kDa | 336_Isiolo | KX368815 |
| 17kDa | 338_Isiolo | KX368816 |
| 17kDa | 339_Isiolo | KX368817 |
| 17kDa | 441_Rachuonyo | KX368818 |
| 17kDa | 442_Bomet | KX368819 |
| 17kDa | 574_Wajir | KX368820 |
| 17kDa | 575_Wajir | KX368821 |
| 17kDa | 577_Wajir | KX368822 |
|  |  |  |
| *ompB* | 044_Wajir | KX368823 |
| *ompB* | 045_Wajir | KX368824 |
| *ompB* | 048_Wajir | KX368825 |
| *ompB* | 051_Moyale | KX368826 |
| *ompB* | 053_Moyale | KX368827 |
| *ompB* | 058_Moyale | KX368828 |
| *ompB* | 060_Moyale | KX368829 |
| *ompB* | 065_Moyale | KX368830 |
| *ompB* | 104_Taita | KX368831 |
| *ompB* | 106_Taita | KX368832 |
| *ompB* | 116_Nyandarua | KX368833 |
| *ompB* | 117_Nyandarua | KX368834 |
| *ompB* | 119_Nyandarua | KX368835 |
| *ompB* | 124_Nyandarua | KX368836 |
| *ompB* | 125_Nyandarua | KX368837 |
| *ompB* | 126_Nyandarua | KX368838 |
| *ompB* | 135_Moyale | KX368839 |
| *ompB* | 136_Moyale | KX368840 |
| *ompB* | 138_Moyale | KX368841 |
| *ompB* | 139_Moyale | KX368842 |
| *ompB* | 140_Moyale | KX368843 |
| *ompB* | 143_Moyale | KX368844 |
| *ompB* | 147_Nanyuki | KX368845 |
| *ompB* | 153_Kajiado | KX368846 |
| *ompB* | 176_Moyale | KX368847 |
| *ompB* | 195_Machakos | KX368848 |
| *ompB* | 256_Mwingi | KX368849 |
| *ompB* | 258_Mwingi | KX368850 |
| *ompB* | 259_Mwingi | KX368851 |
| *ompB* | 260_Kajiado | KX368852 |
| *ompB* | 272_Kajiado | KX368853 |
| *ompB* | 273_Kajiado | KX368854 |
| *ompB* | 275_Kajiado | KX368855 |
| *ompB* | 278_Uasingishu | KX368856 |
| *ompB* | 280_Uasingishu | KX368857 |
| *ompB* | 291_Migori | KX368858 |
| *ompB* | 293_Migori | KX368859 |
| *ompB* | 336_Isiolo | KX368860 |
| *ompB* | 338_Isiolo | KX368861 |
| *ompB* | 339_Isiolo | KX368862 |
| *ompB* | 441_Rachuonyo | KX368863 |
| *ompB* | 442_Bomet | KX368864 |
| *ompB* | 574_Wajir | KX368865 |
| *ompB* | 575_Wajir | KX368866 |
| *ompB* | 577_Wajir | KX368867 |
|  |  |  |
| *ompA* | 044_Wajir | KX368868 |
| *ompA* | 045_Wajir | KX368869 |
| *ompA* | 048_Wajir | KX368870 |
| *ompA* | 051_Moyale | KX368871 |
| *ompA* | 053_Moyale | KX368872 |
| *ompA* | 058_Moyale | KX368873 |
| *ompA* | 060_Moyale | KX368874 |
| *ompA* | 065_Moyale | KX368875 |
| *ompA* | 104_Taita | KX368876 |
| *ompA* | 106_Taita | KX368877 |
| *ompA* | 116_Nyandarua | KX368878 |
| *ompA* | 117_Nyandarua | KX368879 |
| *ompA* | 119_Nyandarua | KX368880 |
| *ompA* | 124_Nyandarua | KX368881 |
| *ompA* | 125_Nyandarua | KX368882 |
| *ompA* | 126_Nyandarua | KX368883 |
| *ompA* | 135_Moyale | KX368884 |
| *ompA* | 136_Moyale | KX368885 |
| *ompA* | 138_Moyale | KX368886 |
| *ompA* | 139_Moyale | KX368887 |
| *ompA* | 140_Moyale | KX368888 |
| *ompA* | 141_Moyale | KX368889 |
| *ompA* | 143_Moyale | KX368890 |
| *ompA* | 144_Moyale | KX368891 |
| *ompA* | 147_Nanyuki | KX368892 |
| *ompA* | 153_Kajiado | KX368893 |
| *ompA* | 164_Wajir | KX368894 |
| *ompA* | 176_Moyale | KX368895 |
| *ompA* | 195_Machakos | KX368896 |
| *ompA* | 241_Mwingi | KX368897 |
| *ompA* | 243_Mwingi | KX368898 |
| *ompA* | 245_Mwingi | KX368899 |
| *ompA* | 247_Mwingi | KX368900 |
| *ompA* | 248_Mwingi | KX368901 |
| *ompA* | 249_Mwingi | KX368902 |
| *ompA* | 250_Mwingi | KX368903 |
| *ompA* | 252_Mwingi | KX368904 |
| *ompA* | 255_Mwingi | KX368905 |
| *ompA* | 256_Mwingi | KX368906 |
| *ompA* | 258_Mwingi | KX368907 |
| *ompA* | 259_Mwingi | KX368908 |
| *ompA* | 260_Kajiado | KX368909 |
| *ompA* | 261_Kajiado | KX368910 |
| *ompA* | 272_Kajiado | KX368911 |
| *ompA* | 275_Kajiado | KX368912 |
| *ompA* | 278_Uasingishu | KX368913 |
| *ompA* | 280_Uasingishu | KX368914 |
| *ompA* | 291_Migori | KX368915 |
| *ompA* | 293_Migori | KX368916 |
| *ompA* | 336_Isiolo | KX368917 |
| *ompA* | 338_Isiolo | KX368918 |
| *ompA* | 339_Isiolo_L | KX368919 |
| *ompA* | 441_Rachuonyo | KX368920 |
| *ompA* | 442_Bomet | KX368921 |
| *ompA* | 574_Wajir | KX368922 |
| *ompA* | 575_Wajir | KX368923 |
| *ompA* | 577_Wajir | KX368924 |
|  |  |  |
| *sca4* | 044_Wajir | KX368925 |
| *sca4* | 045_Wajir | KX368926 |
| *sca4* | 051_Moyale | KX368927 |
| *sca4* | 060_Moyale | KX368928 |
| *sca4* | 065_Moyale | KX368929 |
| *sca4* | 104_Taita | KX368930 |
| *sca4* | 106_Taita | KX368931 |
| *sca4* | 116_Nyandarua | KX368932 |
| *sca4* | 117_Nyandarua | KX368933 |
| *sca4* | 135_Moyale | KX368934 |
| *sca4* | 136_Moyale | KX368935 |
| *sca4* | 138_Moyale | KX368936 |
| *sca4* | 139_Moyale | KX368937 |
| *sca4* | 140_Moyale | KX368938 |
| *sca4* | 143_Moyale | KX368939 |
| *sca4* | 144_Moyale | KX368940 |
| *sca4* | 147_Nanyuki | KX368941 |
| *sca4* | 153_Kajiado | KX368942 |
| *sca4* | 164_Wajir | KX368943 |
| *sca4* | 176_Moyale | KX368944 |
| *sca4* | 195_Machakos | KX368945 |
| *sca4* | 241_Mwingi | KX368946 |
| *sca4* | 243_Mwingi | KX368947 |
| *sca4* | 245_Mwingi | KX368948 |
| *sca4* | 247_Mwingi | KX368949 |
| *sca4* | 248_Mwingi | KX368950 |
| *sca4* | 252_Mwingi | KX368951 |
| *sca4* | 255_Mwingi | KX368952 |
| *sca4* | 256_Mwingi | KX368953 |
| *sca4* | 258_Mwingi | KX368954 |
| *sca4* | 259_Mwingi | KX368955 |
| *sca4* | 260_Kajiado | KX368956 |
| *sca4* | 261_Kajiado | KX368957 |
| *sca4* | 278_Uasingishu | KX368958 |
| *sca4* | 293_Migori | KX368959 |
| *sca4* | 336_Isiolo | KX368960 |
| *sca4* | 338_Isiolo | KX368961 |
| *sca4* | 339_Isiolo | KX368962 |
| *sca4* | 441_Rachuonyo | KX368963 |
| *sca4* | 442_Bomet | KX368964 |
