# Supplementary figures and images for: Phylogenetic Variants of Rickettsia africae, and Incidental Identification of "Candidatus Rickettsia Moyalensis" in Kenya
Source: PLoS Negl Trop Dis. 2016 Jul 7;10(7):e0004788. doi: 10.1371/journal.pntd.0004788 (PMC4936727; doi:10.1371/journal.pntd.0004788)

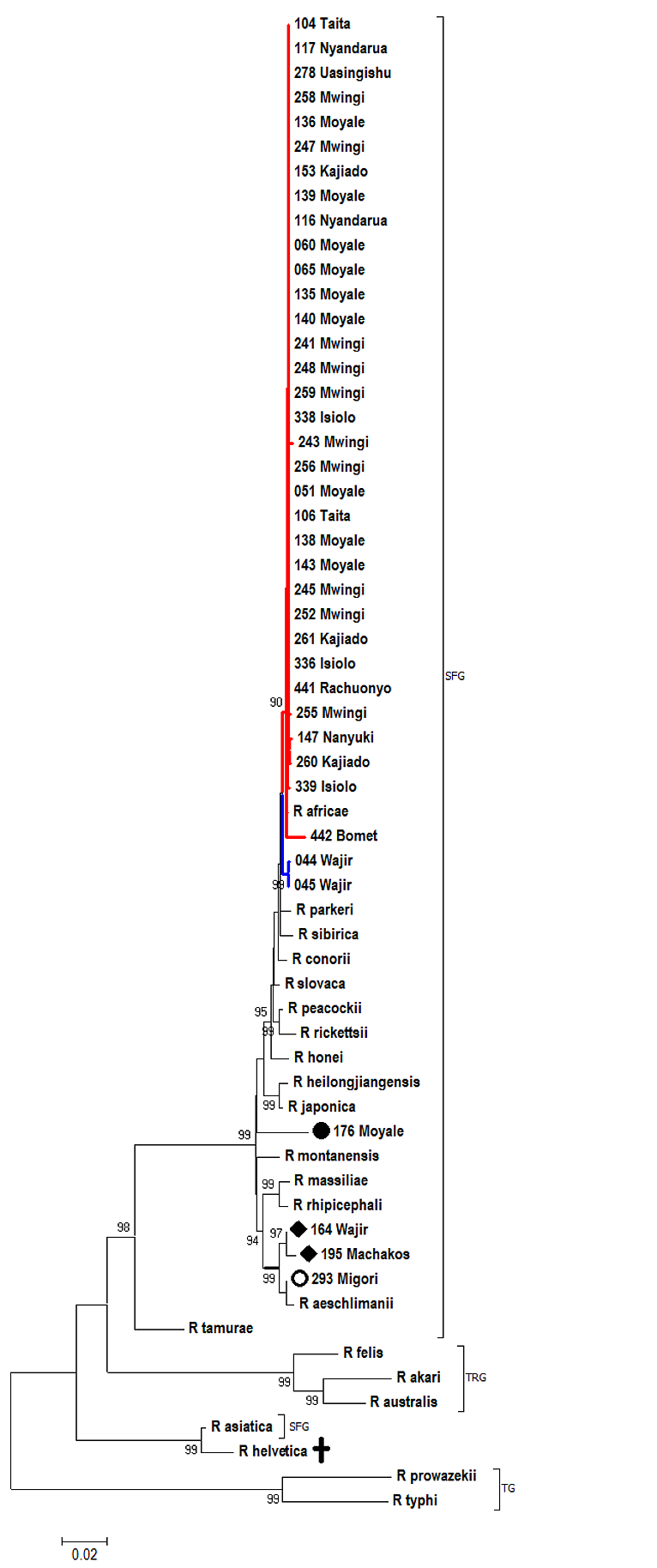

Supplement: S1 Fig — A General Time Reversal with Gamma distribution (GTR+G) model was used to infer phylogeny of concatenated partial sequences of gltA, ompA, ompB, 17kDa and sca4 nucleotide sequences. Amino acid alignments were used to guide the nucleotide alignments. The tree with the highest log likelihood (-8252.6475) is shown. Study samples that are bonafide R. africae aggregate in clades I and II. Samples previously misclassified as R. africae are now classified as R. aeschlimanii (black diamond). Study sample 176_Moyale branches distinctly from other rickettsiae and is considered a novel rickettsia species provisionally named "Candidatus rickettsia moyalensis" (black circle). With this method, 293_Migori (open circle) clusters with R. aeschlimanii. Numbers at the nodes are bootstrap proportions with 1000 replicates. Only bootstrap values >50% are shown. SFG = spotted fever group, TRG = transition group, TG = typhus group. The status of R. helvetica (shown in black cross), originally in spotted fever group is now uncertain [20]. (TIF) [file pntd.0004788.s004.tif]
